# Impact of changes to the interscreening interval and faecal immunochemical test threshold in the national bowel cancer screening programme in England: results from the FIT pilot study

**DOI:** 10.1038/s41416-022-01919-y

**Published:** 2022-08-17

**Authors:** Shuping J. Li, Tara Seedher, Linda D. Sharples, Sally C. Benton, Christopher Mathews, Rhian Gabe, Peter Sasieni, Stephen W. Duffy

**Affiliations:** 1 Wolfson Institute of Population Health, Queen Mary University of London, London, UK; 2 Department of Medical Statistics, London School of Hygiene and Tropical Medicine, London, UK; 3 NHS Bowel Cancer Screening Programme, Southern Hub, Royal County Hospital NHS Foundation Trust, Guildford, Surrey, UK; 4 School of Cancer and Pharmaceutical Sciences, King’s College London, London, UK

**Keywords:** Cancer screening, Cancer screening, Cancer prevention, Cancer prevention, Colorectal cancer

## Abstract

**Introduction:**

The NHS Bowel Cancer Screening Programme (BCSP) faces endoscopy capacity challenges from the COVID-19 pandemic and plans to lower the screening starting age. This may necessitate modifying the interscreening interval or threshold.

**Methods:**

We analysed data from the English Faecal Immunochemical Testing (FIT) pilot, comprising 27,238 individuals aged 59–75, screened for colorectal cancer (CRC) using FIT. We estimated screening sensitivity to CRC, adenomas, advanced adenomas (AA) and mean sojourn time of each pathology by faecal haemoglobin (f-Hb) thresholds, then predicted the detection of these abnormalities by interscreening interval and f-Hb threshold.

**Results:**

Current 2-yearly screening with a f-Hb threshold of 120 μg/g was estimated to generate 16,092 colonoscopies, prevent 186 CRCs, detect 1142 CRCs, 7086 adenomas and 4259 AAs per 100,000 screened over 15 years. A higher threshold at 180 μg/g would reduce required colonoscopies to 11,500, prevent 131 CRCs, detect 1077 CRCs, 4961 adenomas and 3184 AAs. A longer interscreening interval of 3 years would reduce required colonoscopies to 10,283, prevent 126 and detect 909 CRCs, 4796 adenomas and 2986 AAs.

**Conclusion:**

Increasing the f-Hb threshold was estimated to be more efficient than increasing the interscreening interval regarding overall colonoscopies per screen-benefited cancer. Increasing the interval was more efficient regarding colonoscopies per cancer prevented.

## Introduction

Colorectal Cancer (CRC) is the fourth most common cancer in the UK. In 2016–2018, 42,100 CRC diagnoses (19,000 females and 23,900 males) every year contributed to 11% of all new cancer cases. Every year in the UK, around 16,800 bowel cancer deaths occur, equivalent to 46 daily deaths (2017–2019) [1]. Incidence and mortality rates from CRC can potentially be reduced through screening. Faecal testing for blood has been shown to lead to more favourable stage at diagnosis and reduced mortality from the disease, whereas endoscopic screening can detect precancerous adenomas, which can then be removed preventing progression and reducing cancer incidence [2, 3].

The faecal immunochemical test (FIT) quantitates haemoglobin (Hb) in faeces to give a faecal haemoglobin concentration (f-Hb). It has high sensitivity depending on the f-Hb threshold used [4, 5]. In England, FIT was fully adopted in June 2019 as the screening test for CRC and is offered to women and men aged 60–74 years 2-yearly with a positivity threshold of 120 μg/g [6].

Coronavirus disease 19 (COVID-19) is caused by the severe acute respiratory syndrome coronavirus 2 (SARS-CoV-2) [7, 8]. The COVID-19 pandemic has placed considerable strain on healthcare resources [9]. In many areas, including England, cancer screening invitations were suspended due to a lack of available colonoscopy services for those with a positive result [9, 10].

As cancer screening services recover in 2021 and 2022, there are challenges with clearing backlogs generated during the hiatus, and from reduced colonoscopy throughput as a result of measures to minimise the risk of transmission of COVID-19 [9, 11]. The programme is also expanding the age range for FIT testing from 60–74 to 50–74 [12, 13]. Whilst services work hard to clear these backlogs [9, 10, 14], it may be timely to consider potential responses, including a longer interval between screens and/or a higher f-Hb threshold for FIT positivity.

The effect of changes in interscreening interval or f-Hb threshold on-screen detection of early cancer depends on the sensitivity of the test at the chosen threshold, and the mean sojourn time (MST), defined as the average duration of the presymptomatic screen-detectable phase of cancer for that threshold [15].

In this paper, we estimate sensitivity and MST for a range of f-Hb thresholds, and the consequent harvest and prevention of CRC, adenomas, advanced adenomas (AA) and interval cancers (IC) for different combinations of interval and threshold over 15 years of screening. Estimates are derived from the FIT pilot study performed in England in 2014, in which 27,238 persons were screened with FIT [4, 16].

## Methods

### Definition of key terms

- Colonoscopy demand: Under the assumption of 100% uptake, this is assumed to equal the expected number of subjects with positive FIT results.
- Screen-detected CRC: The expected prevalence of CRC at each screening episode.
- Screen-prevented CRC: The expected number of CRC prevented as a result of adenoma excision during a screen, including IC prevented. As some adenomas detected during screening (colonoscopy referral) can progress to CRC if were not excised.
- Screen-benefited CRC: The expected number of CRC benefited from screening in terms of detection or prevention. This equals the sum of screen-detected and screen-prevented CRC.
- Adenomas detected: The sum of high-, intermediate- and low-risk adenomas at each screen episode. The detailed definition was reported previously [16].
- AA detected: The sum of high- and intermediate-risk adenomas at each screening episode. The detailed definition was reported previously [16].
- Interval cancer (IC): The expected number of cancers diagnosed between two screen episodes, excluding the IC prevented from adenoma excision.
- IC prevented: The expected number of IC prevented as a result of adenoma excision during a screen.

### The FIT pilot study

The FIT pilot study has been described in detail previously [4, 16]. In this study, 27,238 participants (14,404 women and 12,834 men) aged 59–75 years in the Southern and Midlands and Northwest regions of England completed a FIT kit (OC-Sensor, Eiken, Japan). Those with an f-Hb of 20 μg/g or more were invited for further diagnostic assessments, usually by colonoscopy. Numbers of participants assessed, numbers of cancers and other abnormalities found by different f-Hb thresholds from 20 upwards have been published [4, 16]. We used the number of positive tests and CRC observed to compare rates of positivity and cancer between screen episodes by logistic regression. In addition, we have estimated sensitivity levels to CRC for a range of f-Hb thresholds [16].

### Statistical estimation

In England, the current bowel screening regimen is to carry out FIT screening with a threshold of 120 μg/g every 2 years [6]. Our aim was to estimate the likely effect, on numbers of screen-detected and prevented cancers, adenomas, AA, and colonoscopies required, of varying the interscreening interval, the f-Hb threshold or both, in response to the current challenges to colonoscopy capacity. All of these outcomes depend on the sensitivity of the test, the interscreening interval and the rate of progression from presymptomatic screen-detectable disease to symptomatic clinical disease. To estimate the expected observed prevalence of adenomas, we already had estimates of sensitivity by threshold (Supplementary Table S2) [16]. We estimated the rate of progression for each threshold using the following assumptions:

- A constant annual incidence of adenomas denoted by I, estimated based on the annual incidence of non-advanced adenomas from Brenner et al.’s paper [17], by sex and age groups between 60 and 74 years old, that is 1930 cases per 100,000 subjects.
  $$I = \frac{{\left( {2.3\% + 2.4\% + 2.2\% } \right) + \left( {1.5\% + 1.65\% + 1.6\% } \right)}}{6} = 0.0193$$
- The screen-detectable phase from cancer first becoming screen-detectable to the onset of symptomatic disease has an exponential distribution with parameter *λ*. The MST is therefore 1/*λ*;
- For a given threshold, there is a constant test sensitivity S to adenomas (using FIT), estimated from the 2014 FIT pilot study [16]; and
- Each test is independent.

The expected observed prevalence of adenoma at the first screen is approximated by

$$P_1 = \frac{{SI}}{\lambda }$$

That is, the product of the mean sojourn time, the sensitivity of the test and the underlying incidence. For further details, see Walter and Day [18] and Michalopoulos and Duffy [19].

At second or subsequent screens, the formula is more complicated. Assume an interscreening interval of *t* years. At a second screen, the expected prevalence of adenoma will be

$$P_2 = S\left\{ {\frac{{\left( {1 - e^{ - \lambda t}} \right)I}}{\lambda } + \frac{{\left( {1 - S} \right)Ie^{ - \lambda t}}}{\lambda }} \right\}$$

where *t* is the interscreening interval. The first component pertains to new adenomas, the second to those missed at the first screen. For a third or later screen, the probability is approximated by

$$P_{3 + } = S\left\{ {\frac{{\left( {1 - e^{ - \lambda t}} \right)I}}{{\lambda \left[ {1 - \left( {1 - S} \right)e^{ - \lambda t}} \right]}}} \right\}$$

This is the limiting form of the expected number when the number of previous tests tends to infinity. These are simplifications of the probabilities in Walter and Day [18, 19]. Since we have estimates of *I* from published data, *S* is known from previous work [16] and *t* is known to be 2 years, we had only one parameter, *λ*, to estimate.

To estimate *λ*, we treated the numbers of adenomas at first, second and later screens as binomial with probabilities *P*_*1*_, *P*_*2*_ and *P*_*3*_, respectively, and estimated *λ* by maximising the product of binomial likelihoods.

Let *n*_*i*_ and *c*_*i*_ be the numbers screened and adenomas detected at screen number *i*. Then, given *n*_*i*_, the number of adenomas detected *c*_*i*_ has a binomial distribution with probability _*Pi*_, and the likelihood is:

$$L = P_1^{c_1}\left( {1 - P_1} \right)^{\left( {n_1 - c_1} \right)}P_2^{c_2}\left( {1 - P_2} \right)^{\left( {n_2 - c_2} \right)}P_3^{c_3}\left( {1 - P_3} \right)^{\left( {n_3 - c_3} \right)}$$

The likelihood was maximised using the Broyden–Fletcher–Goldfarb–Shanno (BFGS) method, a quasi-Newton method. Optimisation of the kernel of the likelihood function was carried out using the ‘optim’ command, in R version 3.4.2 [20–23].

We then used the formulae for *P*_*1*_*, P*_*2*_ and *P*_*3*_ to estimate the likely harvest of adenomas detected (assume 100% removed) at first, second and subsequent screens for thresholds from 20 to 180 μg/g, and interscreening intervals of 1, 2, 3, 4 and 5 years. Finally, for each threshold and interscreening interval combination, we estimate the total number of screen-detected cancers and associated number of colonoscopies per 100,000 screened in a period of 15 years.

We also used the same formulae with different values of incidence and sensitivity to estimate the progression rate, thus the expected prevalence of advanced adenomas. Finally, we estimated the total number of screen-detected and screen-prevented cancers, also the associated number of colonoscopies for 100,000 screened over a period of 15 years. The screen-detected cancers were estimated using the same procedure as for adenomas above, but corrected by subtraction of cancers estimated to be prevented as a result of detection and removal of adenomas. Sections C, D and E in the supplementary material provide full details.

In estimating over the 15-year period, we reduced the population to be screened at each round by the number of AAs and cancers found previously as the number of screening increases. This is based on the current policy that screenees found with AA or cancer are moved to surveillance or treatment, thus excluded from follow-up screenings [24]. A final assumption on estimating the demand on colonoscopy service was to assume for 100% uptake, therefore this equivalates to the number of positive FIT results.

### Adjusting figures for cancers prevented due to adenoma detection

In addition to cancers detected early, some cancers will be prevented as a result of the detection and removal of precedent adenomas. Pinsky et al. [25] estimated in a meta-analysis that the number of adenomas needed to remove (NNR) to prevent one CRC is 52 (95% CI, 36–93), given the time frame used to estimate NNR is 11 years, and the time frame we use is 15 years. Thus, with a simple linear extrapolation, we used NNR at 38 ($52 \times 11 \div 15 = 38$), that is one CRC is prevented for every 38 adenomas removed (see section D in the supplementary material for an example).

### Estimating deaths prevented in 5 years

Further to reducing cancer incidence, screening ultimately translates to improved cancer mortality, namely deaths prevented. Chan et al. [26] found that 5-year survival in screen-detected cancers was 42.5% compared with 36.2% in symptomatic cancers. We calculated 5-year deaths prevented from both aspects of screening—detection and prevention compared with no screening:

1. by screen detection, the number of deaths prevented is 0.063 × *n*, where *n* is the number of screen-detected cancers (since 0.425–0.362 = 0.063);
2. by screen prevention, the number is 0.638 × *m*, where *m* is the number of screen-prevented cancers (1–0.362 = 0.638). This assumes that the cancers prevented would otherwise have been symptomatic.

## Results

During the 2014 FIT pilot study, of the 27,238 participants who completed FIT, 1825 had a f-Hb at 20 μg/g or above and underwent colonoscopy. Most participants had previously responded to a screening invitation (previous responders) (75%, *n* = 20,465), of which 16,355 completed at least two screening rounds prior to the FIT pilot episode (third time or more participants). For 6773 subjects, this was their first bowel screening (first-time participants). Table 1 lists participants’ characteristics by geographical hub, sex, age group and Index of Multiple Deprivation (IMD) quintile.

**Table 1:** Characteristics of populations screened in the FIT pilot study.

	First time (*n*, %)		Second time (*n*, %)		Third time or more (*n*, %)	
Screen episode	6773	–	4110		16,355	–
Hub						
Southern	3651	53.9%	2421	58.9%	8671	53.0%
Midlands and North West	3122	46.1%	1689	41.1%	7684	47.0%
Sex						
Male	3445	50.9%	1910	46.5%	7479	45.7%
Female	3328	49.1%	2200	53.5%	8876	54.3%
Age group (years)						
59–64	5187	76.6%	3168	77.1%	2799	17.1%
65–69	952	14.1%	180	4.4%	8553	52.3%
70–75	634	9.4%	762	18.5%	5003	30.6%
IMD quintile						
IMD 1	977	14.4%	414	10.1%	1625	9.9%
IMD 2	1060	15.7%	627	15.3%	2394	14.6%
IMD 3	1480	21.9%	907	22.1%	3496	21.4%
IMD 4	1582	23.4%	1015	24.7%	4089	25.0%
IMD 5	1673	24.7%	1145	27.9%	4750	29.0%
IMD n/k*	1	<0.1%	2	<0.1%	1	<0.1%

*Participants where postcode could not be linked to layer super output areas (LSOA).

IMD, index of multiple deprivation, IMD 1 to IMD 5 is a scale from the most to least deprived.

First- and second-time participants were younger, with 77% being under 65 years old, compared to only 17% of third time or more participants. Across all screening episodes, more participants were from the Southern hub than the Midlands and North West hub and uptake increased with higher IMD classification.

Supplementary Table S1 gives the observed number of positive screens and cancers detected from 27,238 participants in the FIT pilot study, by screening episodes and f-Hb thresholds. At a threshold of 20 μg/g, the number of positive tests across different episodes was similar (7.8–8.0%), however, at thresholds of 40 μg/g or more, first-time participants had a higher number of positives than previous responders. Adjusting for threshold, the rates for both positivity and cancer detection reduce significantly at later screens (*P* < 0.001) in both cases.

While a lower threshold implied a higher proportion of tests with a positive result (positivity rate) and a better cancer detection rate, doubling the positivity rate (colonoscopies) did not guarantee a doubled cancer detection rate. The combined (across all screen episodes) positivity rate for a threshold of 80 μg/g was double that for a threshold of 180 μg/g (2.9% vs 1.5%), while the combined cancer detection rate increased only by 46% (0.19% vs 0.13%). At the current screening threshold of 120 μg/g only a quarter of participants would be referred, compared to that from a threshold of 20 μg/g (2.1% vs 7.8%), but more than half of cancers would be detected (43 vs 74 cancers).

Supplementary Table S2 shows the estimated sensitivity from Li et al. [16], estimated MST and progression rate from presymptomatic screen-detectable phase to symptomatic disease for CRC, AA and adenomas. At 120 μg/g, the sensitivity to CRC was estimated as 47.8% with 3.37 years MST (95% CI: 2.52–5.12 years). Sensitivity dropped with each incremental increase in f-Hb threshold and was below 50% for thresholds of 120 μg/g or above. Conversely, estimated MSTs of CRC (i.e. the time to progress from presymptomatic screen-detectable to symptomatic disease that is picked up clinically) were similar across all thresholds, all between 3 and 4 years. The estimated sensitivity of FIT to AA at 120 µg/g was just below a quarter at 23% with MST at 5.26 years. Sensitivity was estimated to be above 50% only for the low threshold at 20 µg/g and it decreased steeply to 16.22% at 180 µg/g. The corresponding MST ranged from 7.18 to 5.13 years.

Table 2 shows the estimated numbers of colonoscopies (positive FIT results), CRC, AA and adenomas detected and IC prevented by screening in 100,000 subjects over 15 years, by interscreening interval and f-Hb threshold, as well as estimated deaths prevented in the five years following diagnosis, from each combination. Under the current strategy of 2-yearly screening and 120 μg/g positivity threshold, screening 100,000 subjects would incur 16,092 colonoscopies, and detect 1142 CRC over a period of 15 years (8 screening rounds). Thus, with the current screening policy, we detect one cancer for every 14.1 colonoscopies and prevent one cancer for every 86.3 colonoscopies (Table 3). While a lower threshold implies better cancer prevention and greater cancer death prevention, it places substantial demand on colonoscopy services. For 2-yearly screening, a very low threshold of 20 μg/g would nearly triple the number of cancers prevented and detect 2.27 times more AA compared to a threshold of 120 µg/g. However, it would require 3.7 times more colonoscopies and would detect only one cancer per 48 colonoscopies and prevent one cancer per 107 colonoscopies. On the basis of guidelines, we would expect that each CRC detected would generate two follow-up colonoscopies, however, these would take place in any case, albeit later, when the CRC was detected symptomatically, or at a subsequent screen. We would, however, expect that each advanced adenoma would generate at least one further colonoscopy which would not otherwise have taken place [27]. Thus, from the detection of AA, the number of colonoscopies would increase by around 20% for 1–2-year intervals and by 25–30% for 3–5-year intervals (Table 2). Total colonoscopies, including these follow-up examinations, are given in Supplementary Table S3.

**Table 2:** Estimated demand on colonoscopy (positive FIT), screening benefits including screen-detected and screen-prevented CRC, interval cancers prevented, adenomas and AA detected and deaths prevented per 100,000 screened in a 15-year period, by f-Hb thresholds (μg/g) and interscreening intervals (years).

Threshold	Interscreening interval	Colonoscopy demand	Screen-detected CRC	Screen-prevented CRC	AA detected	Adenomas detected	IC prevented	Deaths prevented*
20	1	108,131	1317	658	11,830	25,006	91	503
	2	58,812	1216	550	9671	20,894	137	427
	3	37,391	1130	429	7720	16,315	146	345
	4	30,151	1060	383	7185	14,550	160	311
	5	22,877	958	313	6120	11,889	150	260
40	1	72,864	1385	528	10,373	20,066	82	424
	2	39,595	1246	408	7944	15,488	113	339
	3	25,112	1118	302	6098	11,472	113	263
	4	20,219	1028	261	5523	9935	118	232
	5	15,295	904	208	4577	7912	108	190
80	1	41,254	1432	374	7992	14,222	74	329
	2	22,448	1212	260	5594	9887	89	242
	3	14,254	1019	182	4060	6902	81	180
	4	11,491	906	153	3542	5800	81	154
	5	8710	762	119	2835	4507	70	124
120^†^	1	29,431	1432	286	6451	10,857	70	272
	**2**	**16,092**	**1142**	**186**	**4259**	**7086**	**76**	**191**
	3	10,283	909	126	2986	4796	65	138
	4	8327	784	105	2549	3972	62	116
	5	6360	635	80	2003	3053	53	91
150	1	24,642	1437	243	5591	9249	64	246
	2	13,495	1119	154	3590	5857	66	169
	3	8644	871	103	2476	3911	56	121
	4	7013	743	85	2091	3219	53	101
	5	5373	592	65	1629	2463	44	79
180	1	21,021	1427	211	5071	8015	62	224
	2	11,500	1077	131	3184	4961	61	151
	3	7360	816	86	2170	3281	50	106
	4	5968	686	71	1821	2688	46	88
	5	4570	537	658	1412	2050	38	68

*FIT* faecal immunochemical test, *CRC* colorectal cancer, *AA* advanced adenomas, *IC* interval cancer, *f-Hb* faecal haemoglobin concentration.

^†^Estimates for the current screening policy are in bold.

*Deaths prevented for 5 years following diagnosis.

**Table 3:** Estimated positive predicted value and number needed for colonoscopy for CRC, AA and adenomas by f-Hb thresholds (μg/g) and interscreening intervals (years).

Threshold	Interscreening interval	Screen-detected CRC		Screen-prevented CRC		Screen-benefited CRC		AA detected		Adenomas	
		PPV	NNC	PPV	NNC	PPV	NNC	PPV	NNC	PPV	NNC
20	1	1.22%	82.1	0.61%	164.3	1.83%	54.7	10.94%	9.1	23.13%	4.3
	2	2.07%	48.4	0.93%	107.0	3.00%	33.3	16.44%	6.1	35.53%	2.8
	3	3.02%	33.1	1.15%	87.1	4.17%	24.0	20.65%	4.8	43.63%	2.3
	4	3.52%	28.4	1.27%	78.7	4.79%	20.9	23.83%	4.2	48.26%	2.1
	5	4.19%	23.9	1.37%	73.1	5.56%	18.0	26.75%	3.7	51.97%	1.9
40	1	1.90%	52.6	0.72%	138.0	2.63%	38.1	14.24%	7.0	27.54%	3.6
	2	3.15%	31.8	1.03%	97.1	4.18%	23.9	20.06%	5.0	39.12%	2.6
	3	4.45%	22.5	1.20%	83.2	5.65%	17.7	24.28%	4.1	45.68%	2.2
	4	5.08%	19.7	1.29%	77.3	6.38%	15.7	27.32%	3.7	49.14%	2.0
	5	5.91%	16.9	1.36%	73.5	7.27%	13.7	29.93%	3.3	51.73%	1.9
80	1	3.47%	28.8	0.91%	110.2	4.38%	22.8	19.37%	5.2	34.48%	2.9
	2	5.40%	18.5	1.16%	86.3	6.56%	15.2	24.92%	4.0	44.04%	2.3
	3	7.15%	14.0	1.27%	78.5	8.42%	11.9	28.48%	3.5	48.42%	2.1
	4	7.88%	12.7	1.33%	75.3	9.21%	10.9	30.83%	3.2	50.48%	2.0
	5	8.75%	11.4	1.36%	73.4	10.11%	9.9	32.55%	3.1	51.75%	1.9
120*	1	4.86%	20.6	0.97%	103.0	5.84%	17.1	21.92%	4.6	36.89%	2.7
	**2**	**7.10%**	**14.1**	**1.16%**	**86.3**	**8.25%**	**12.1**	**26.47%**	**3.8**	**44.03%**	**2.3**
	3	8.84%	11.3	1.23%	81.5	10.06%	9.9	29.03%	3.4	46.65%	2.1
	4	9.42%	10.6	1.26%	79.7	10.67%	9.4	30.61%	3.3	47.71%	2.1
	5	9.98%	10.0	1.26%	79.2	11.24%	8.9	31.49%	3.2	48.00%	2.1
150	1	5.83%	17.1	0.99%	101.2	6.82%	14.7	22.69%	4.4	37.53%	2.7
	2	8.29%	12.1	1.14%	87.6	9.43%	10.6	26.61%	3.8	43.40%	2.3
	3	10.08%	9.9	1.19%	84.0	11.27%	8.9	28.64%	3.5	45.25%	2.2
	4	10.60%	9.4	1.21%	82.8	11.81%	8.5	29.81%	3.4	45.91%	2.2
	5	11.03%	9.1	1.21%	82.9	12.23%	8.2	30.33%	3.3	45.84%	2.2
180	1	6.79%	14.7	1.00%	99.7	7.79%	12.8	24.12%	4.1	38.13%	2.6
	2	9.37%	10.7	1.14%	88.1	10.50%	9.5	27.69%	3.6	43.14%	2.3
	3	11.09%	9.0	1.17%	85.2	12.26%	8.2	29.48%	3.4	44.58%	2.2
	4	11.50%	8.7	1.19%	84.4	12.69%	7.9	30.51%	3.3	45.04%	2.2
	5	11.74%	8.5	1.18%	84.7	12.92%	7.7	30.90%	3.2	44.86%	2.2

*CRC* colorectal cancer, *AA* advanced adenomas, high-risk and intermediate-risk adenomas combined, *f-Hb* faecal haemoglobin concentration, *PPV* positive predictive value, *NNC* number needed to colonoscopy.

*Results for the current screening policy are in bold.

Increasing the interscreening interval and/or raising positivity thresholds was estimated to reduce the requirement for colonoscopy and decrease CRC detection. A one-third reduction in colonoscopies can be achieved by either raising the interscreening interval to every 3 years or by raising the threshold to 180 μg/g. At the cost of reducing CRC detection by ~20% and 6%, and prevented deaths by 28% and 21%, respectively. However, both strategies achieve a better colonoscopy cancer detected ratio than the current policy (11.3 and 10.7 vs 14.1). In contrast, raising the threshold from 120 to 150 μg/g was estimated to reduce required colonoscopies by ~16% without substantially impacting CRC detection (16,092 vs 13,495 colonoscopies and 1142 vs 1119 CRC detected, for 120 g/g and 150 μg/g, respectively) (Table 3). In terms of colonoscopies per cancer prevented as a result of adenoma detection, relaxing the interscreening interval would appear to be more efficient (Table 3). The current policy is estimated to require 86.3 colonoscopies per cancer prevented. The corresponding figures for 2-yearly screening with a threshold of 180 μg/g and 3-yearly screening with a threshold of 120 μg/g would be 88.1 and 81.5, respectively.

Increasing the interscreening interval and/or raising positivity thresholds was also estimated to decrease the detection of adenomas and AA. Compared to screening 2-yearly at 120 µg/g, screening 3-yearly at the same threshold was estimated to reduce adenomas and AA detection by 32% and 30%, respectively. Similar impacts were estimated for 2-yearly screening at threshold 180 µg/g, with estimated reductions of 30% and 25%.

## Discussion

We used estimates of screening sensitivity and sojourn time from the English FIT pilot study to predict the likely effects of changes to the English bowel cancer screening programme, which might be considered as possible actions to address challenges faced due to COVID-19, such as the screening backlog and reduced colonoscopy service caused by new safety measures [9, 11, 14]. We estimated the impact on colonoscopy services and CRC detection over a period of 15 years, by varying interscreening interval and/or f-Hb threshold.

Currently, the English CRC programme’s policy is to screen 2-yearly with f-Hb positivity threshold of 120 μg/g. This has an estimated sensitivity to CRC of 47.8% with 3.37 years MST (95% CI: 2.52–5.12 years) (Supplementary Table S2), and is estimated to benefit 1328 subjects (detect 1142 CRC and prevent 186 CRC), and 4259 subjects in terms of AA detected by carrying out 16,092 colonoscopies for every 100,000 subjects screened over a 15-year period (colonoscopy cancer benefited ratio of 12.1) (Tables 2 and 3).

Our results can be used to inform strategies to relax the current policy, in order to address limitations in capacity due to the COVID-19 pandemic [9], or to expand the screening to a lower starting age. Policy decisions will depend on the trade-off between the reduction in the colonoscopy rate and the resulting numbers of cancers missed or delayed. For example, if the strategy is primarily based on a reduction in colonoscopy demand per cancer missed, then increasing the threshold to 150 μg/g (113 colonoscopies avoided per cancer missed) or 180 μg/g (71 colonoscopies avoided per cancer missed), while maintaining a 2-year interval, would be reasonable options. Alternatively, to avoid 5000 or more colonoscopies, viable options would be to either increase the threshold to 180 μg/g without changing the interscreening interval, or move to screening every three years with the current threshold of 120 μg/g. Both policies have a better colonoscopy per cancer benefited ratio. However, compared to the current policy, we would miss an additional 6% or 20% of cancers detected (1077 and 909 vs 1142), prevent 30% or 32% fewer cancers (131 and 126 vs 186), at the same time increase expected IC by 14% or 34% (977 and 1150 vs 856), and prevent 21% and 28% fewer deaths (151 and 138 vs 191), respectively (Tables 2, 3 and S3 in the supplementary).

Our analysis has several strengths. First, data were from a population-based screening programme for average-risk individuals in England, so that results are generalisable to the target population for screening. Second, to estimate the MST for CRC, we used sensitivity estimates of gFOBT to CRC from Kearns et al. [28], to model cancers missed at the gFOBT screen which preceded the FIT screen in the UK pilot (in the projections of results of repeated FIT screening, of course we used the sensitivity of FIT for each threshold). Third, using empirically estimated MST, we derived screen-detected cancers, prevented cancers (due to excision of screen-detected adenomas), adenomas and AA detected, and interval cancers (cancers diagnosed between screenings) for a range of interscreening intervals and f-Hb thresholds. These provide potentially useful information to inform decisions about potential immediate changes to the NHS Bowel Cancer Screening Programme in response to the COVID-19 pandemic, and to cope with an increased screening population in the future.

There are some limitations, notably the modelling assumptions we made, including that of a constant underlying incidence of preclinical cancer and a constant progression rate from presymptomatic to symptomatic disease, λ, over a 15-year period, and by implication a 15-year age range. Both assumptions are consistent with existing findings. For example, Soriano et al. found that the CRC incidence remained relatively stable in the UK over the last decade [29]. Though colorectal cancer incidence does increase with age [15], the underlying incidence rate used in our estimates covers the majority (77%) of the FIT pilot study cohort. For the second assumption, Chiu et al. found the use of a constant *λ* in an exponential model to be a good fit for modelling the MST of CRC [30]. In addition, derived estimates were consistent with published findings [31].

When estimating the required number of colonoscopies in Table 2, we assumed that the number of screen positives depended only on the threshold, and not on the interval. This might overestimate the number of colonoscopies generated by annual screening, and underestimate the number of colonoscopies for interscreening intervals longer than 2 years. Also, note that the estimated demand on colonoscopies assumed for 100% uptake is likely to differ in actual screening. In the UK FIT pilot, the colonoscopy uptake rate varied from 79.84% to 87.26%, depending on gender and threshold. There was no clear trend in uptake with threshold, and the average uptake was 82.28%. If we consider that all the benefit in terms of adenoma removal and cancer detection occurs in those who have a colonoscopy, it is reasonable to make the approximation that the number of colonoscopies and all benefits in terms of early detection and prevention would be diluted to 82.28% of those reported above [4].

**Fig. 1:**
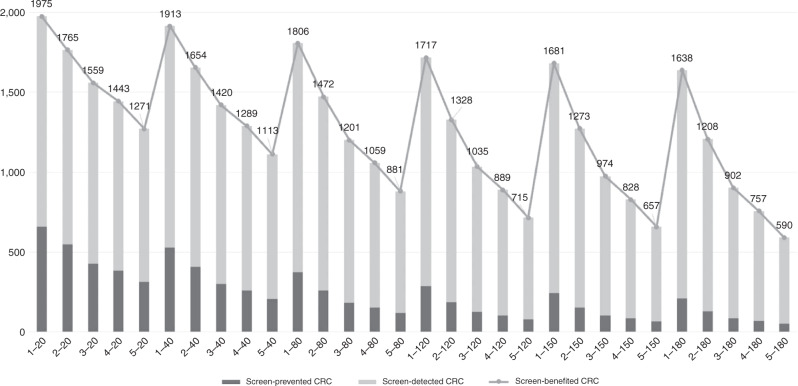
Estimated number of cancers benefited from screening by interscreening interval (years) and f-Hb threshold (µg/g). The horizontal axis gives the combination of interscreening interval in years and faecal haemoglobin threshold in µg/g. The vertical axis shows the total number benefited from screening in terms of cancer, this is the point labelled atop the stacked bars for the given combination, made up of cancers prevented (darker) and detected (lighter) from screening. Take the first bar as an example, for every 100,000 individuals over 15 years, ‘1–20’ means to screen every year with a threshold at 20 µg/g, 1975 is the estimated total number of individuals benefited from screening regarding cancer, made up by 658 from prevention and 1317 from detection.

Further, the imposition of a fixed period of screening, to reflect the age range of screening of 60–74 years, has implications for the effectiveness of the interval. For example, for an interscreening interval of 4 years, the estimated number of colonoscopies and screen-detected cancers over 15 years is in fact only calculated for up to 13 years (the subsequent round is in the 17th year), and similarly, for the number of adenomas, AA and IC expected. The same issue underlies the observation that estimates all appear much lower for an interscreening interval of 5 years, as this implies three screens with the last at 70 years old (Fig. 1). Another notable restriction was that the numbers of deaths prevented were estimated for only 5 years following diagnosis, whereas results of screening trials suggest that prevention of deaths would continue for a longer period of follow-up. Thus the numbers of prevented deaths are underestimated.

We also estimated sensitivity first, then conditioned MST on sensitivity, as we were restricted by the small number of cancers observed (74 cancers) in the FIT pilot study. This small number precluded the use of more complex models to estimate the sensitivity and MST simultaneously or to estimate statistics by CRC stages. However, the estimates we obtained were consistent with published studies [32]. One further caveat applies, although lengthening the interscreening interval or raising the f-Hb threshold will reduce the number of colonoscopies overall, the exact number of colonoscopies generated by symptomatic presentation between screens is unclear. We estimated that raising the threshold to 120 µg/g at 3-yearly screening would incur 18% more expected IC compared to the screening at 180 µg/g with a less frequent 2-yearly interval. While not all missed lesions or CRCs will result in an interval CRC, one would expect more symptomatic presentations with a longer interscreening interval, and for participants of female gender and older age [14, 33]. If we assume, for example, each AA requires at least one further follow-up colonoscopy, then raising the threshold to 180 µg/g requires 198 more colonoscopies than continuing screening at 120 µg/g with a less frequent 3-yearly interval over 15 years. Lastly, if an abnormality only bleeds up to a certain level below the threshold adopted then it may not be detected at screening regardless of the interscreening interval of FIT.

To address concerns that current referrals would be denied colonoscopy if a higher threshold was adopted, a stratified approach may ensure an acceptable compromise between risks and benefits [16, 34, 35]. For example:

- f-Hb <120 μg/g: repeat FIT in 3 years; [36, 37],
- f-Hb 120–180 μg/g: repeat FIT in 6 months. Colonoscopy only if repeated FIT result ≥180 μg/g; [38] and
- f-Hb ≥180 μg/g: colonoscopy.

Note that we are not explicitly recommending this strategy or these actions. This is simply an example of the approach one might take. The repeated use of FIT, a home testing kit, may better identify at-risk individuals with fewer hospital visits, ensuring that limited colonoscopy and wider health service is directed towards those in greatest need. More data are needed to ascertain the safety and effectiveness of such an approach.

The capacity issue is the major challenge in restoring and improving the English Bowel Cancer Screening Programme. In future, the NHS plans to reduce the lower age limit for FIT to 50 years and to use a threshold that is more sensitive to both cancer and adenomas [9, 12, 13]. In the short term, however, compromises in the threshold and frequency of screening may be required. Both may result in missing cancers, increased numbers of IC and potentially lead to less favourable outcomes. Raising the threshold reduces referrals for colonoscopy, but increases the chance of false negative results, delaying treatment to cancer or adenomas, while lengthening the interval reduces the chance of testing while the tumour is in the preclinical phase. If such decisions are necessary, our results provide an evidence base for policymakers to minimise the effects of increasing demand and/or restrictions in capacity.

In conclusion, circumstances may dictate that one cannot have both the optimal interscreening interval and the optimal threshold. Relaxing at least one of these can relieve pressure on the healthcare system in the short term. Raising the f-Hb threshold to 180 μg/g was estimated to reduce the required number of colonoscopies by a third, with only a 6% reduction in CRC detection over a 15-year period. A stratified approach to management may provide a more acceptable compromise.

### Reporting summary

Further information on research design is available in the Nature Research Reporting Summary linked to this article.

## Supplementary information

Supplementary

Reporting Summary

## Data Availability

Requests for data should be sent to NHS Digital. The authors do not have the authority to share the data.
