# Supplementary material for: Impact of changes to the interscreening interval and faecal immunochemical test threshold in the national bowel cancer screening programme in England: results from the FIT pilot study
Source: Br J Cancer. 2022 Aug 17;127(8):1525–33. doi: 10.1038/s41416-022-01919-y (PMC9553931; doi:10.1038/s41416-022-01919-y)
Supplement: Supplementary file 1 — Supplementary [file 41416_2022_1919_MOESM1_ESM.pdf]

**Supplementary material**

**Contents**


**A. Supplementary Tables**

**Table S1:** Observed number and rate of positive screens, CRC, AA and adenomas detected by screening episode, f-Hb thresholds (μg/g) in the FIT pilot study.

**Table S2:** Estimates of sensitivity and mean sojourn time (years) to symptomatic CRC, AA and adenomas by f-Hb thresholds (μg/g).

**Table S3:** Estimated number of interval cancers expected and colonoscopies for per 100,000 screened in a 15-year period, by f-Hb thresholds (μg/g) and interscreening intervals (years).

**B. Formulae used in estimation**

1. Positive FIT (colonoscopy demand)
2. CRC
3. Adenomas detected
4. AA detected
5. IC

Figure S1: Relationship between screening key term

**C. Further details of estimating the likely harvest of screen detected cancers**

**Table S4:** Comparison of estimated number of screen-detected CRC by subtraction and formula for per 100,000 screened in a 15-year period, by f-Hb thresholds (μg/g) and interscreening intervals (years).

**D. Further details of estimating numbers of cancers prevented as a result of adenoma detection and excision**

**E. Further details of estimating numbers of advanced adenomas and CRC detected**

**F. Further details of estimating numbers of interval cancers (IC)**

**G. Example calculation**

**References**

## A. Supplementary Tables

**Table S1.** Observed number and rate of positive screens, CRC, AA and adenomas detected by screening episode, f-Hb thresholds (µg/g) in the FIT pilot study.

| Threshold | Screening episode* | Positive | Positivity rate | Cancer | Cancer detection rate | AA | AA detection rate | Adenoma | Adenoma detection rate |
|---|---|---|---|---|---|---|---|---|---|
| **20** | **1** | 541 | 8.0% | 21 | 0.31% | 128 | 1.89% | 237 | 3.50% |
| | **2** | 319 | 7.8% | 11 | 0.27% | 69 | 1.68% | 134 | 3.26% |
| | **3+** | 1,273 | 7.8% | 42 | 0.26% | 280 | 1.71% | 576 | 3.52% |
| | **Combined** | 2,133 | 7.8% | 74 | 0.27% | 477 | 1.75% | 947 | 3.48% |
| **40** | **1** | 365 | 5.4% | 19 | 0.28% | 95 | 1.40% | 164 | 2.42% |
| | **2** | 207 | 5.0% | 10 | 0.24% | 47 | 1.14% | 87 | 2.12% |
| | **3+** | 849 | 5.2% | 37 | 0.23% | 214 | 1.31% | 401 | 2.45% |
| | **Combined** | 1,421 | 5.2% | 66 | 0.24% | 356 | 1.31% | 652 | 2.39% |
| **80** | **1** | 221 | 3.3% | 14 | 0.21% | 68 | 1.00% | 107 | 1.58% |
| | **2** | 110 | 2.7% | 9 | 0.22% | 27 | 0.66% | 55 | 1.34% |
| | **3+** | 471 | 2.9% | 30 | 0.18% | 132 | 0.81% | 221 | 1.35% |
| | **Combined** | 802 | 2.9% | 53 | 0.19% | 227 | 0.83% | 383 | 1.41% |
| **120** | **1** | 166 | 2.5% | 13 | 0.19% | 51 | 0.75% | 82 | 1.21% |
| | **2** | 80 | 1.9% | 7 | 0.17% | 17 | 0.41% | 28 | 0.68% |
| | **3+** | 330 | 2.0% | 23 | 0.14% | 97 | 0.59% | 154 | 0.94% |
| | **Combined** | 576 | 2.1% | 43 | 0.16% | 165 | 0.61% | 264 | 0.97% |
| **150** | **1** | 141 | 2.1% | 12 | 0.18% | 46 | 0.68% | 68 | 1.00% |
| | **2** | 68 | 1.7% | 7 | 0.17% | 12 | 0.29% | 20 | 0.49% |
| | **3+** | 274 | 1.7% | 21 | 0.13% | 78 | 0.48% | 127 | 0.78% |
| | **Combined** | 483 | 1.8% | 40 | 0.15% | 136 | 0.50% | 215 | 0.79% |
| **180** | **1** | 121 | 1.8% | 12 | 0.18% | 42 | 0.62% | 58 | 0.86% |
| | **2** | 57 | 1.4% | 6 | 0.15% | 12 | 0.29% | 19 | 0.46% |
| | **3+** | 233 | 1.4% | 18 | 0.11% | 65 | 0.40% | 103 | 0.63% |
| | **Combined** | 411 | 1.5% | 36 | 0.13% | 119 | 0.44% | 180 | 0.66% |

CRC: colorectal cancer; AA: advanced adenomas, high-risk and intermediate-risk adenomas combined; f-Hb: faecal haemoglobin concentration; FIT: faecal immunochemical test.

* 1, 2, 3+ means a participant takes a FIT for the first time, second time, or third time or more.

Total number screened by FIT kits is 27,238 in the pilot study.

**Table S2** Estimates of sensitivity of FIT and mean sojourn time (years) to symptomatic CRC, AA and adenomas by f-Hb thresholds (µg/g).

| | **Colorectal cancer** | | | **Advanced adenomas** | | | **Adenomas** | | |
|---|---|---|---|---|---|---|---|---|---|
| **f-Hb threshold in µg/g** | **S** | **MST (95% CI)** | **λ (95% CI)** | **S** | **MST (95% CI)** | **λ (95% CI)** | **S** | **MST (95% CI)** | **λ (95% CI)** |
| **20** | 82.2% | 3.97 (3.15-5.38) | 0.25 (0.19-0.32) | 63.30% | 7.18(6.40-8.18) | 0.14(0.12-0.16) | 41.26% | 5.93 (5.50-6.42) | 0.17(0.16-0.18) |
| **40** | 73.3% | 3.86 (3.02-5.34) | 0.26 (0.19-0.33) | 46.68% | 6.71(5.94-7.71) | 0.15(0.13-0.17) | 27.95% | 5.48 (5.04-6.00) | 0.18(0.17-0.20) |
| **80** | 58.9% | 3.60 (2.75-5.21) | 0.28 (0.19-0.36) | 30.85% | 5.77(5.02-6.78) | 0.17(0.15-0.20) | 17.10% | 4.85(4.38-5.43) | 0.21(0.18-0.23) |
| **120** | 47.8% | 3.37 (2.52-5.12) | 0.30 (0.20-0.40) | 23.01% | 5.26(4.50-6.32) | 0.19(0.16-0.22) | 12.25% | 4.49(3.98-5.14) | 0.22(0.19-0.25) |
| **150** | 44.4% | 3.29 (2.43-5.08) | 0.30 (0.20-0.41) | 19.02% | 5.09(4.31-6.22) | 0.20(0.16-0.23) | 9.94% | 4.43(3.89-5.14) | 0.23(0.19-0.26) |
| **180** | 40.0% | 3.17 (2.32-5.04) | 0.32 (0.20-0.43) | 16.22% | 5.13(4.30-6.34) | 0.20(0.16-0.23) | 8.33% | 4.37(3.80-5.15) | 0.23(0.19-0.26) |

***S: sensitivity; MST: Mean sojourn time; f-Hb: faecal haemoglobin concentration. All estimated using interscreening interval at 2 years.**

**Table S3**. Estimated number of interval cancers expected and colonoscopies for per 100,000 screened in a 15-year period, by f-Hb thresholds (µg/g) and interscreening intervals (years).

| Threshold | Interscreening Interval | Interval cancers | Total colonoscopies |
|---|---|---|---|
| **20** | 1 | 210 | 119,961 |
| | 2 | 419 | 68,483 |
| | 3 | 625 | 45,111 |
| | 4 | 741 | 37,337 |
| | 5 | 913 | 28,997 |
| **40** | 1 | 271 | 83,236 |
| | 2 | 531 | 47,539 |
| | 3 | 765 | 31,210 |
| | 4 | 895 | 25,742 |
| | 5 | 1,072 | 19,873 |
| **80** | 1 | 378 | 49,246 |
| | 2 | 713 | 28,042 |
| | 3 | 984 | 18,315 |
| | 4 | 1,126 | 15,033 |
| | 5 | 1,304 | 11,545 |
| **120†** | 1 | 467 | 35,881 |
| | **2** | **856** | **20,351** |
| | 3 | 1,150 | 13,268 |
| | 4 | 1,296 | 10,875 |
| | 5 | 1,470 | 8,363 |
| **150** | 1 | 504 | 30,233 |
| | 2 | 912 | 17,085 |
| | 3 | 1,211 | 11,120 |
| | 4 | 1,357 | 9,104 |
| | 5 | 1,527 | 7,003 |
| **180** | 1 | 547 | 26,092 |
| | 2 | 977 | 14,684 |
| | 3 | 1,282 | 9,530 |
| | 4 | 1,427 | 7,789 |
| | 5 | 1,594 | 5,982 |

Estimated interval cancers are corrected for cancers prevented through screening. Total number of colonoscopies includes one additional colonoscopy per AA detected. † Estimates for the current screening policy are in bold.

**B: Formulae used in estimation**

1. **Positive FIT (colonoscopy) each round**

We assume 100% uptake for referrals, so that the required number of colonoscopies equals the number of positive FIT results in each screening round.

Positive FIT = population screened x positivity rate [a]

[a] Positivity rate based on observed values from the 2014 FIT pilot study, separated by 1st time screen, 2nd time screen, and subsequent screens. (Table S1)

2. **CRC**

- Screen-detected CRC (by subtraction) = total CRC with the screening – screen-prevented CRC

- Screen-detected CRC (by formula) = population screened x expected observed prevalence of CRC

See the main text methods section and section C in supplementary material for more details.

- Screen-prevented CRC due to adenoma excision = adenoma excision prevented CRC/ number of adenomas needed to remove to prevent one CRC

See section D and E in supplementary material for further details.

- Screen-benefited CRC = screen-detected CRC + screen-prevented CRC

3. **Adenomas detected**

Adenomas detected from each round = population screened x expected observed prevalence of adenoma

See the main text methods section for further details.

4. **Advanced adenomas (AA) detected**

AA detected from each round = population screened x the expected prevalence of advanced adenoma.

See section E in supplementary materials for further details.

5. **Interval cancers (IC)**

- IC after nth screen = population screened at n+1th screen x probability of IC between nth and n+1th screen

- IC prevented (from adenoma excision) = Screen-prevented CRC due to adenoma excision x probability the prevented CRC is an IC

- IC expected after nth screen = IC after nth screen – IC prevented

See section F in supplementary materials for further details.

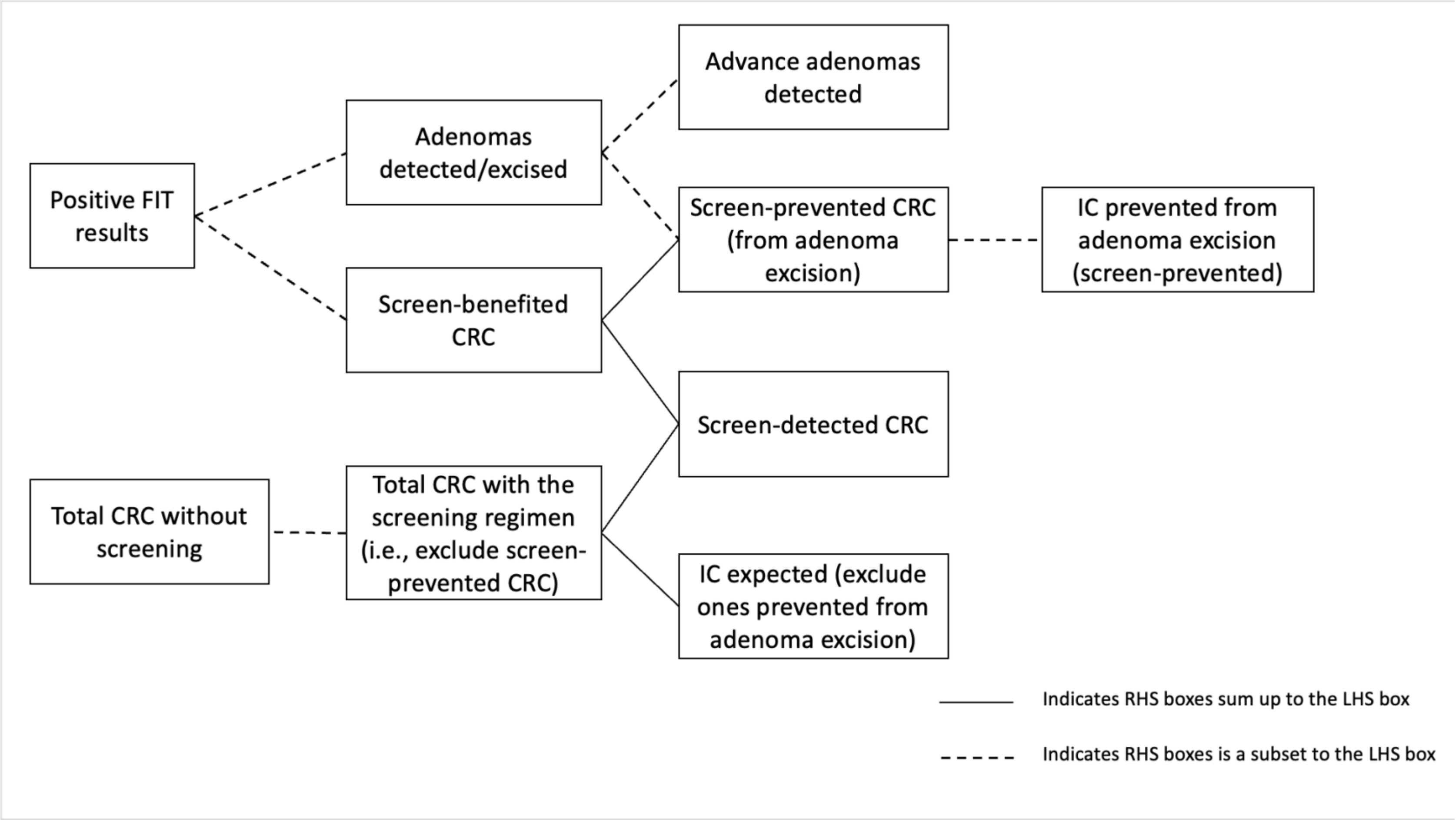


*Figure S2 Relationship between screening key terms*

**C. Further details of estimating the likely harvest of screen detected cancers**

In the main text, the expected screen-detected CRC were calculated by subtracting screen-prevented CRC from the total screen-detected CRC expected for a given screening regimen. The expected observed prevalence of CRC can also be calculated based on the same formula as that for adenomas (formulae for $P_1$, $P_2$, and $P_3$ in the main text), with different values for incidence, progression rate and sensitivity:

- a constant annual incidence of preclinical screen-detectable cancer denoted by I, estimated by the age-and-sex standardised incidence rate of CRC for age group 60 to 69 years in 2004, just before the screening programme started. That was 145.64 cases per 100,000 subjects (142.22, 149.13); [6]
- the time to progression from the presymptomatic screen-detectable phase to symptomatic disease has an exponential distribution with parameter λ. The MST is therefore 1/λ;
- for a given threshold there is a constant test sensitivity S (using FIT); and
- each test is independent.

In the Faecal Immunochemical Test (FIT) pilot, this was the participants' first screen using FIT. Previous screens were done using the guaiac Faecal Occult Blood Test (gFOBT). To estimate cancers missed from previous screening in likelihood estimation, we used gFOBT sensitivity estimates to CRC from Kearns et al, that is 27.35% (95% CI: 26.84%, 27.87%) when used in the prevalent round (the first screen), and then 20.22% (95% CI: 19.53%, 20.93%) in incident rounds (repeated screens). [1] To estimate cancers missed from the pilot study episode, we used the estimated sensitivity of FIT to CRC from previous work (Table S2), [2] and assumed this to be constant for all episodes for a given threshold. To incorporate varied test sensitivities in estimating the total number of screen-detected cancers, for a given interscreening interval *t* at a given threshold, the expected observed prevalence of cancer at each screen episode is as follows.

Prevalence at 1$^{st}$ screen:

$$P_1 = \frac{S_1 I}{\lambda}$$

Prevalence at 2$^{nd}$ screen:

$$P_2 = \frac{S_2 I}{\lambda}\left[\left(1 - e^{-\lambda t}\right) + (1 - S_1)e^{-\lambda t}\right]$$

Prevalence at 3$^{rd}$ screen:

$$P_3 = \frac{IS_3}{\lambda}[\left(1-e^{-\lambda t}\right)+(1-S_2)\left(1-e^{-\lambda t}\right)e^{-\lambda t}+(1-S_1)(1-S_2)e^{-2\lambda t}]$$

Where $S_1$ and $S_2$ are sensitivity to CRC using gFOBT in the prevalent and incident episodes respectively, and $S_3$ is the sensitivity to CRC using FIT. *I* is the constant annual incidence of preclinical screen-detectable cancer in the population, and $\lambda$ is the parameter of the exponentially distributed mean sojourn time of colorectal cancer.

Similar to calculating the expected prevalence of adenomas, substituting the appropriate values for I and S, we first optimised the kernel of each likelihood function to estimate $\lambda$, then calculate the expected prevalence of CRC at first, second and subsequent screens using formulae $P_1$, $P_2$, and $P_3$, and finally estimated the number of CRC to be detected in varied combinations of threshold and inerscreening interval. (Table S4)

**Table S4** Comparison of estimated number of screen-detected CRC by subtraction and formula for per 100,000 screened in a 15-year period, by f-Hb thresholds (µg/g) and interscreening intervals (years).

| **Threshold** | **Interscreening Interval** | **By subtraction*** | **By formula*** | **Difference** |
|---|---|---|---|---|
| **20** | 1 | 1,317 | 1,635 | 318 |
| | 2 | 1,216 | 1,524 | 308 |
| | 3 | 1,130 | 1,152 | 21 |
| | 4 | 1,060 | 1,065 | 4 |
| | 5 | 958 | 912 | -46 |
| **40** | 1 | 1,385 | 1,724 | 339 |
| | 2 | 1,246 | 1,537 | 291 |
| | 3 | 1,118 | 1,169 | 52 |
| | 4 | 1,028 | 1,062 | 34 |
| | 5 | 904 | 888 | -16 |
| **80** | 1 | 1,432 | 1,674 | 242 |
| | 2 | 1,212 | 1,397 | 185 |
| | 3 | 1,019 | 1,043 | 24 |
| | 4 | 906 | 923 | 17 |
| | 5 | 762 | 749 | -13 |
| **120†** | 1 | 1,432 | 1,530 | 99 |
| | **2** | 1,142 | 1,206 | 64 |
| | 3 | 909 | 878 | -31 |
| | 4 | 784 | 761 | -23 |
| | 5 | 635 | 606 | -29 |
| **150** | 1 | 1,437 | 1,477 | 39 |
| | 2 | 1,119 | 1,140 | 21 |
| | 3 | 871 | 823 | -48 |
| | 4 | 743 | 709 | -35 |
| | 5 | 592 | 561 | -32 |
| **180** | 1 | 1,427 | 1,385 | -42 |
| | 2 | 1,077 | 1,040 | -37 |
| | 3 | 816 | 741 | -75 |
| | 4 | 686 | 633 | -54 |
| | 5 | 537 | 497 | -39 |

* As shown in Table 2 in the main text.

## D. Further details of estimating numbers of cancers prevented as a result of adenoma detection and excision

### Cancer prevented from adenoma excision

Pinsky et al[5] estimated in a meta-analysis that the number of adenomas needed to remove (NNR) to prevent one CRC is 52 (95% CI, 36-93), given the time frame used to estimate NNR is 11 years, and the time frame we use is 15 years. Thus, with a simple linear extrapolation, we used NNR at 38 ( $52 \times 11 \div 15 = 38$), that is one CRC is prevented for every 38 adenomas removed.

Consider the case of a threshold of 20 µg/g and a one-year interval, if we detect 25,006 adenomas over 15 years for per 100,000 screened, then the total number of cancers prevented from adenoma excision would be 658.

## E. Further details of estimating numbers of advanced adenomas and CRC detected

### Advanced adenomas

Again, the expected prevalence of advanced adenoma is based on the same formula as that for CRC ($P_1$, $P_2$, and $P_3$ in the main text), with different values for incidence, progression rate and sensitivity:

- A constant annual incidence of advanced adenoma denoted by I, estimated based on Brenner et al.[3, 4] Using $\lambda_1$- the weighted annual incidence rate of adenomas at 1930 cases per 100,000 subjects,[4] and $\lambda_2$ - the annual transition rate from advanced adenoma to CRC at 0.03916,[3] this would give the annual incidence as 0.6%.

$$\lambda_1 = \frac{(2.3\% + 2.4\% + 2.2\%) + (1.5\% + 1.65\% + 1.6\%)}{6} = 0.0193$$

$$\lambda_2 = \frac{3.1\% + 3.8\% + 5.1\% + 2.7\% + 3.8\% + 5.0\%}{6} = 0.03916$$

To estimate the annual incidence of advanced adenoma, let us assume that in a given year, any AA's were born as non-advanced adenomas either in that year or in the nine years preceding. Thus, incidence of AA's in a year would be:

$$\int_0^9 \lambda_1 e^{-\lambda_1 t} e^{-\lambda_2 (9-t)} dt\left(1 - e^{-\lambda_2}\right) + \int_0^1 \lambda_1 e^{-\lambda_1 t}(1 - e^{-\lambda_2 (1-t)} dt$$

Integrating out, this is:

$$\frac{\lambda_1(1 - e^{-\lambda_2})(e^{-9\lambda_1} - e^{-9\lambda_2})}{\lambda_2 - \lambda_1} + 1 - e^{-\lambda_1} - \frac{\lambda_1(e^{-\lambda_1} - e^{-\lambda_2})}{\lambda_2 - \lambda_1}$$

- screen-detectable phase to symptomatic disease has an exponential distribution with parameter λ. The MST is therefore 1/λ;
- For a given threshold there is a constant test sensitivity S to AA (using FIT); and
- Each test is independent.

Similar to calculating the expected prevalence of CRC, using the threshold dependent sensitivity of FIT to advanced adenoma estimated, we optimised the kernel of each likelihood function and then calculated the expected prevalence of adenoma by threshold and inerscreening interval.

## F. Further details of estimating numbers of interval cancers (IC)

### IC total

We can predict the probability of interval cancers following a screen for an interval t, as follows. Following the first screen, the probability of a person having an interval cancer before the next screen t years later is:

$$\int_0^t I \int_0^{t-s} \lambda e^{-\lambda u} du ds + \frac{(1-S)I(1-e^{-\lambda t})}{\lambda}$$

The first component represents the probability of a new cancer being born and then progressing to symptoms within the interval. The second represents a cancer being missed at the original screen and progressing to symptoms in the interval. This simplifies to

$$\frac{I}{\lambda}\left\{\lambda t - \left(1-e^{-\lambda t}\right) + (1-S)(1-e^{-\lambda t})\right\}$$

Following second or subsequent screens (remember our simplifying assumption that a cancer is not missed twice), the probability is

$$\int_0^t I \int_0^{t-s} \lambda e^{-\lambda u} du ds + \frac{(1-S)I(1-e^{-\lambda t})(1-e^{-\lambda t})}{\lambda}$$

Again, the first component is the probability of a new cancer being born and progressing to symptoms in the interval, the second the probability of a cancer missed at the previous round becoming symptomatic in the interval. This simplifies to:

$$\frac{I}{\lambda}\left\{\lambda t - \left(1-e^{-\lambda t}\right) + (1-S)(1-e^{-\lambda t})^2\right\}$$

the estimates we already have of I, λ and S for CRC, we can calculate the expected interval cancer probabilities for a 1, 2, 3, 4 and 5 year interval for the various thresholds.

### IC prevented

Using similar logic to previously, if $\lambda_1$ is the progression rate from adenoma to asymptomatic cancer and $\lambda_2$ the progression rate from asymptomatic to symptomatic cancer, then for a given adenoma present at a screen, the probability of it arising as an interval cancer prior to the next screen is:

$$a = 1 - e^{-\lambda_1 t} - \frac{\lambda_1(e^{-\lambda_1 t} - e^{-\lambda_2 t})}{\lambda_2 - \lambda_1}$$

The probability of it being detected at the next screen is

$$b = \frac{S\lambda_1(e^{-\lambda_1 t} - e^{-\lambda_2 t})}{\lambda_2 - \lambda_1}$$

Therefore we can approximate the proportion of prevented cancers that are interval cancers as

$$P_I = \frac{a}{a+b}$$

For a given interval and threshold we know S, $\lambda_2$ and t. From Pinsky's et al, [5] if 1 in 52 adenomas becomes a cancer in 11 years, then we have

$$\int_0^{11} e^{-\lambda_1 t} dt = 1/52$$

This solves to give $\lambda_1$= 0.0018.

Consider again the case of a threshold of 20 µg/g and a one-year interval, for per 100,000 screened over 15 years, it is estimated to detect 25,006 adenomas and prevent 658 cancers from adenoma excision, (Section D)

Take t=1, $\lambda_1$= 0.0018 and $\lambda_2$ = 0.2516 (Table S2), we have a= 0.0002085 and b=0.0013069, and the IC prevented as a proportion of prevented cancers is $P_1$=0.1376. Thus, the expected number of IC prevented from adenoma excision is:

$$IC\ prevented = 0.1376 \times 658 = 91$$

And the total number of IC expected excluding the IC prevented from adenoma excision is:

$$IC\ expected = 300 - 91 = 209$$

## G. Example

Based on the incidence of CRC from the National Cancer Registry [6] (section C, supplementary), that is 145.64 cases per 100,000 subjects, we expect 2185 CRCs over a 15-year period without screening. **Consider the case of a threshold of 20 μg/g and a one-year interval**, based on the observed positivity rates in the FIT pilot study, we expect 108,131 positive FIT results, and the same for colonoscopy demand assuming for 100% uptake.

Based on the formula for expected prevalence of adenomas and AA, with a screening regimen of the above, we expect to detect 25,006 adenomas and 11,830 AA. Assume for 100% adenoma excision during colonoscopy and that Pinsky et al's [5] estimation that 1 CRC is prevented for every 38 adenomas removed, screen-prevented CRC due to adenoma excision is expected to be 658, of which, 91 would have arisen as an IC between screening rounds.

Thus the total number of CRC expected to occur excluding those which would have been prevented is (2185-658=)1527, and this further splits into screen-detected CRC and IC expected. The latter is calculated in section F, that is 209 IC expected, and the screen-detected CRC is (1527-209=)1318.

To estimate the number of deaths prevented 5-year following the diagnosis, we sum the estimated deaths prevented from screening detection and prevention using formulae from the methods section in the main paper. The deaths prevented from screen-detection are ($0.063 \times 1317=$) 83, and from screen-prevention are ($0.638 \times 658=$) 420 respectively. Thus, the total number of deaths prevented from screening is (83+420=)503.

**References**

1. Kearns B, Whyte S, Chilcott J, Patnick J. Guaiac faecal occult blood test performance at initial and repeat screens in the English Bowel Cancer Screening Programme. Br J Cancer. 2014;111(9):1734- 41.

2. Li SJ, Sharples LD, Benton SC, Blyuss O, Mathews C, Sasieni P, Duffy SW. Faecal immunochemical testing in bowel cancer screening: Estimating outcomes for different diagnostic policies. J Med Screen. 2020 Dec 20:969141320980501.

3. Brenner H, Altenhofen L, Stock C, Hoffmeister M. Natural history of colorectal adenomas: birth cohort analysis among 3.6 million participants of screening colonoscopy. Cancer Epidemiology and Prevention Biomarkers. 2013 Jun 1;22(6):1043-51.

4. Brenner H, Altenhofen L, Stock C, Hoffmeister M. Incidence of colorectal adenomas: birth cohort analysis among 4.3 million participants of screening colonoscopy. Cancer Epidemiology and Prevention Biomarkers. 2014 Sep 1;23(9):1920-7.

5. Pinsky PF, Loberg M, Senore C, Wooldrage K, Atkin W, Bretthauer M, Cross AJ, Hoff G, Holme O, Kalager M, Segnan N. Number of adenomas removed and colorectal cancers prevented in randomized trials of flexible sigmoidoscopy screening. Gastroenterology. 2018 Oct 1;155(4):1059-68.

6. National Cancer Registration & Analysis Service, Public Health England. Cancer Data (snapshot reference CAS1902) [Internet]; June 2018 [cited 15th August 2020]. Available from: https://www.cancerdata.nhs.uk/incidence/age_standardised_rates
